# Targeted dosing for susceptible heteroresistant subpopulations may improve rational dosage regimen prediction for colistin in broiler chickens

**DOI:** 10.1038/s41598-023-39727-w

**Published:** 2023-08-07

**Authors:** Andrew Mead, Pierre-Louis Toutain, Pascal Richez, Ludovic Pelligand

**Affiliations:** 1 Comparative Biomedical Sciences, The Royal Veterinary College, London, UK; 2 Transpharm, Saint-Genies des Mourgues, France; 3 INTHERES, Université de Toulouse, INRAE, ENVT, Toulouse, France

**Keywords:** Microbiology, Antimicrobials, Therapeutics

## Abstract

The dosage of colistin for the treatment of enteric *E. coli* in animals necessitates considering the heteroresistant (HR) nature of the targeted inoculum, described by the presence of a major susceptible population (S1, representing 99.95% of total population) mixed with an initial minor subpopulation of less susceptible bacteria (S2). Herein, we report the 1-compartment population pharmacokinetics (PK) of colistin in chicken intestine (jejunum and ileum) and combined it with a previously established pharmacodynamic (PD) model of HR in *E. coli.* We then computed probabilities of target attainment (PTA) with a pharmacodynamic target (AUC_24h_/MIC) that achieves 50% of the maximal kill of bacterial populations (considering inoculums of pure S1, S2 or HR mixture of S1 + S2). For an MIC of 1 mg/L, PTA > 95% was achieved with the registered dose (75,000 IU/kg BW/day in drinking water) for the HR mixture of S1 + S2 *E. coli*, whether they harboured *mcr* or not. For an MIC of 2 mg/L (ECOFF), we predicted PTA > 90% against the dominant susceptible sub-population (S1) with this clinical dose given (i) over 24 h for *mcr*-negative isolates or (ii) over 6 h for *mcr*-positive isolates (pulse dosing). Colistin clinical breakpoint S ≤ 2 mg/L (EUCAST rules) should be confirmed clinically.

## Introduction

Colistin is used in chickens for the treatment and metaphylaxis (following confirmation of disease in flock) of susceptible enteric *E. coli* infections. The dose recommended for administration via drinking water is 75,000 IU of colistin per kg body weight, per day, for 3–5 consecutive days in Europe^1^. This form of administration also leads to whole-flock treatment in which the dose is directly related to the amount of water consumed per kg bodyweight, resulting in varied and potentially suboptimal dosage in a percentage of animals. As colistin is poorly absorbed from the gastrointestinal tract (with negligible bioavailability in plasma), its effect is exerted locally within the intestinal lumen of the intestine. Subsequently, the colistin concentration–time profile must be focussed on the gastrointestinal tract (topical effect), and not serum concentrations. Furthermore, this focus rests on the small intestine (jejunum and ileum) representing the principle compartment for potentially pathogenic *E. coli* (as compared to the large intestine). Gastrointestinal content of colistin has been previously reported for gavage administration of high doses (500,000 and 1,000,000 IU/kg BW^2^) and following the clinical dosing regimen of 75,000 IU/kg BW/day over 3 days^3^. No data is available on the colistin content reached in the gastro intestinal tract (GIT) with alternative dosage regimens (higher doses, gavage or pulse dosing). PK models predicting gut concentrations of antimicrobial drug (AMD) have been proposed for various species, using empirical data, between the gut and circulation^4–6^ and then in more detail regarding the physiological flow between gut segments^7,8^. Collection of digestive content in small species, such as chickens at a young age, is challenging as their size precludes the placement of invasive intestinal dialysis probes for repeated sampling^9^, leading to destructive sampling methods. Furthermore, a faecal sampling alternative is flawed due to the mixture of faecal and urine material in birds. Therefore, knowledge of the gut flow and content around the circadian cycle is required to discuss the local disposition of colistin in the GIT and to develop an ad hoc PK model describing the fate of colistin after a semi-continuous oral administration in water, especially when individual birds contribute to only a single sample.

Chicken gut physiology is complex, with birds GIT evolved to reduce the energetic cost of flying through the shortening of the gastrointestinal tract (compared to mammals) and the subsequent transit time^10^. In addition, the photoperiodic ingestive behaviour of poultry precludes the prediction of how long an orally administered AMD would be retained in the different segment of the GIT following a discontinuous pattern of ingestion^11^. Numerous studies have explored the mean residence time (MRT), using various tracers (physical or chemical markers), that indicate short transit times between 2.8 h and 5.6 h after food intake^12–16^. Multiple factors may affect this estimation, including the granularity of feed, type of tracer, bird age and lineage. More importantly, during non-feeding times (i.e. the period of darkness or scotoperiod), the transit slows down^17^, which is associated with local stagnation of the digestive contents^18^ and therefore, with a probable continuity of local exposure to colistin despite the cessation of colistin ingestion. Further complicating this physiological retention is the presence of multiple specialised compartments (e.g. crop, proventriculus, gizzard, duodenum, etc.) with different functions^19,20^, two-large valved caeca that may retain digesta for more than 24 h and retro-peristaltic movements that optimise digestion^21–23^. Generally, the efficacy of an antimicrobial can only be ensured if the free concentration remains above the minimum inhibitory concentration (MIC) of the susceptible bacteria (either %T > MIC or AUC/MIC)^24,25^ although colistin shows a concentration dependent bias which indicates efficacy of colistin in the GIT may be associated with higher concentrations for shorter time periods. Furthermore, *E. coli* exhibits a heteroresistant response when exposed to colistin, i.e. the total initial population comprises a dominant susceptible population (estimated at 99.95%) and a less-susceptible sub-dominant (estimated at 0.05%) population^26^. This may suggest that targeting the dominant (S1) population may be sufficient to achieve a significant reduction in the total number of enteric *E. coli.* As such, there is a need to develop an intestinal PK and PK/PD models to predict the gut concentration and effect throughout the entire nychthemeron for different modalities of colistin oral ingestion at different dosage regimen (typically over 3–5 days, with 3 days being the current trend to limit antibiotic usage and optimise dosage regimen), where birds moderate their feeding and drinking behaviour through increased intake activity in anticipation of the night period which alters the content weight and passage rate of liquids and feed.

This study aimed to (i) measure the intestinal colistin content reached during and after a 72 h oral administration via drinking water in broiler chickens at the current clinical dose of 75,000 IU/kg (approved in all EU countries for poultry), supra-clinical doses of 100,000 IU/kg and 150,000 IU/kg, and as single oral-gavage doses at 75,000 IU/kg and 150,000 IU/kg, (ii) develop an intestinal PK model from measure colistin concentration in the GIT to predict the colistin exposure through Monte-Carlo Simulation (MCS), (iii) determine the nature and critical value of the PK/PD index (using in silico dose fractionation), and (iv) use the validated simple model to simulate alternative dosage regimens such as pulse-dosing and compute probabilities of target attainment (PTA).

## Results

### Back-calculation of dose administered

**Figure 1:**
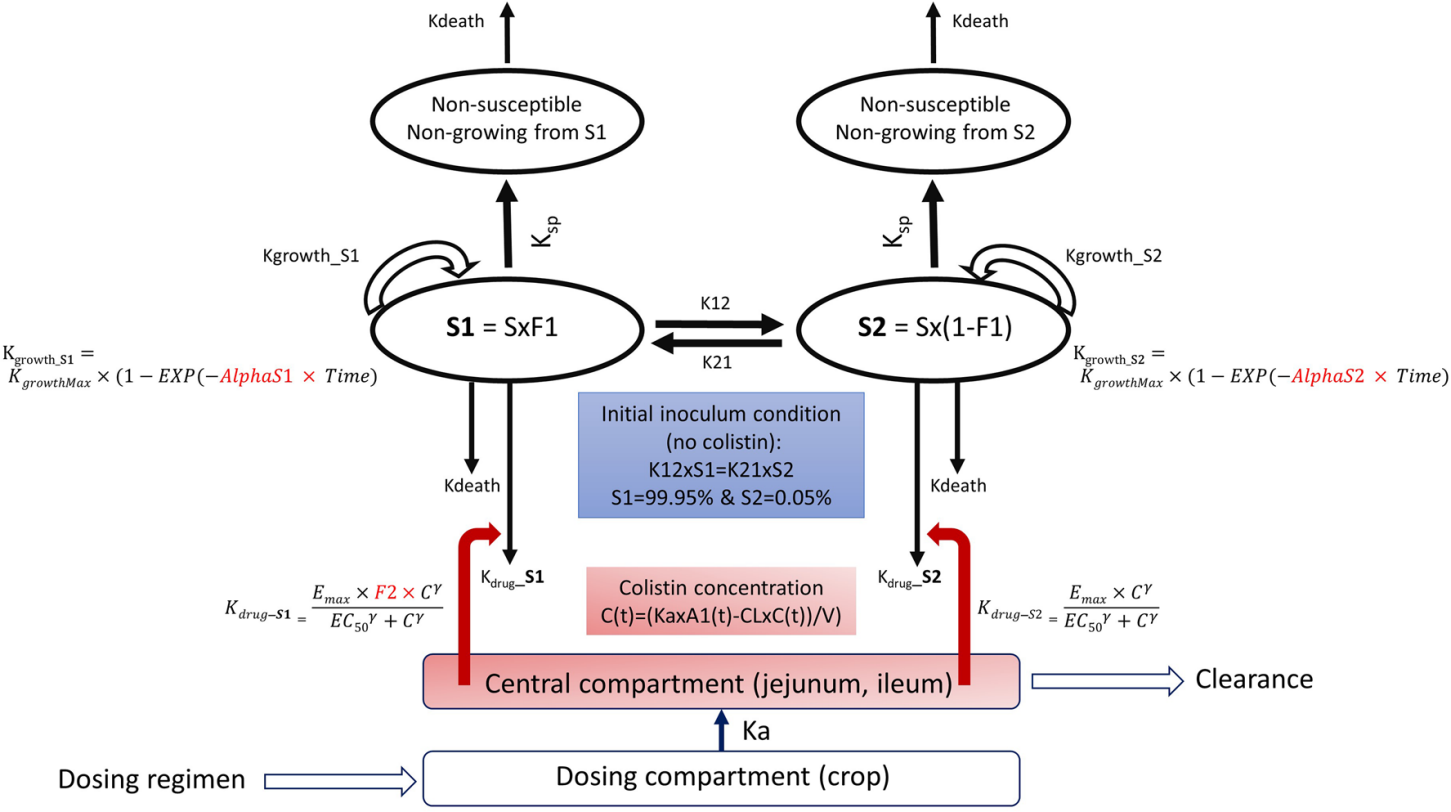
Diagrammatic representation of the PK/PD model. A simple 1-compartment pharmacokinetic model describes the transit (ka) of colistin to the small intestine (jejunum and ileum) from the crop with the concentration (C) defined by the amount of colistin (A), volume of distribution (V) and clearance (Cl), a flow representing excretion via the colon. Colistin concentration in the small intestine is applied to the pharmacodynamic heteroresistance model^26^ and to each of the sub-populations (S1 dominant susceptible population; S2 sub-dominant less-susceptible). In this PD model the fraction for each sub-population is determined by a distribution factor (F1) controlled by K12 and K21. At time 0 (no colistin), most of the inoculum is in the S1 fraction, all populations have an equal irreversible transfer rate to a non-growing state (K_SP_) and a constant rate of natural cell death (K_death_) fixed to 0.17 h^−1^. The colistin concentration informs K_drug_ that is considered as additive to the natural cell death, with a potentiation factor (F-2) for the highly susceptible subpopulation S1. Probability of target attainment is determined for the total population (S1 + S2) or each fractional sub-population independently.

Chicken (13 days old) were administered colistin via oral gavage (75,000 and 150,000 IU/kg) directly into the crop or in drinking water for 3 days at nominal doses of 75,000, 100,000 and 150,000 IU/kg BW/day (adjusted for colistin base potency of 30,000 IU/mg) and actual doses, back calculated based on measured water consumption for each 24 h period, were 78,023, 97,667, and 141,133 IU/kg BW/day, respectively (Supplementary Table S1). The overarching PK/PD model (Fig. 1) highlights the route of administration through the crop to the intestinal compartment.

### Pharmacokinetics of colistin in chickens

**Figure 2:**
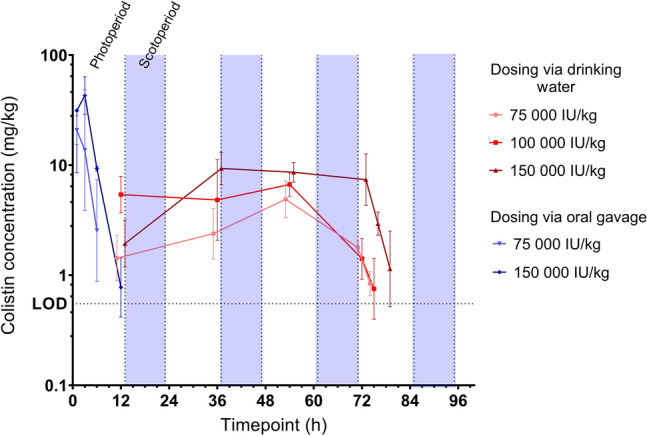
Geometric mean (± geometric SD) of colistin intestinal content (mg/kg) after administration of 75,000 IU/kg BW/day via drinking water (n = 38), 100,000 IU/kg BW/day via drinking water (n = 38), 150,000 IU/kg BW/day via drinking water (n = 38) and 75,000 IU/kg BW oral gavage (n = 17), 150,000 IU/kg oral gavage (n = 17). Limit of detection (LOD) is 0.55 mg/kg. Photoperiod is time exposed to light (day); scotoperiod the time in absence of light (night).

Colistin concentration in luminal intestinal (jejunum and ileum) content (LIC), following administration via drinking water, was measurable from the first time point (12 h post-onset of dosing) and was detectable throughout the dosing period. Geometric mean concentrations were 4.89 mg/kg, 6.66 mg/kg, and 8.60 mg/kg at 54 h for doses of 75,000 IU/kg, 100,000 IU/kg, and 150,000 IU/kg respectively. Oral gavage achieved considerably higher LIC immediately following dosing of 21.25 mg/kg after 1 h for 75,000 IU/kg dose and 45.55 mg/kg after 3 h for 150,000 IU/kg dose (Fig. 2).

### Compartmental modelling

A simple 1-compartment model with administration either via bolus gavage or continuous zero-order administration via dosing compartment (crop) and sampling from small intestine was fitted and its PK parameters are reported in Table 1. The entry rate constant from the crop to jejunum was estimated at 0.221 h^−1^ (yielding a 3.14 h entry rate half-life or 4.5 h mean transit time). Intestinal “clearance” was estimated to 0.291 kg content/kg BW/h, i.e. 29.1 g intestinal content per kg BW per hour, for intestinal “volume” estimated to 5.14 g intestinal content per kg BW.

**Table 1:** Pharmacokinetic parameter estimates (typical values) for the 1-compartment colistin intestinal PK model.

Parameter	Estimate (between subject variability %)	Units	CV%	2.5% CI	97.5% CI
Primary parameters					
ka	0.221	h^−1^	21.3	0.13	0.32
V	5.14	g intestinal content/kg BW	74.4	− 2.4	12.7
Cl	29.1 (40.5%)	g intestinal content/kg BW/h	6.3	25.5 (27%)	32.7 (51.2%)
Multiplicative residual error	36.2	%	6.9	31.2	41.2
Secondary parameters					
ka HL	3.14	h	21.3	1.80	4.45
Overall MTT in digestive tract	4.5	h	–		

Entry rate constant (ka), volume of distribution (V), clearance (Cl), half-life (HL), and Mean Transit Time (MTT), which is the average time a colistin molecule takes to transit between the crop and the jejunum. CV% and confidence interval (CI) giving precision of estimate.

Equation (1) defines the daily total dose of colistin transited through the small intestine as calculated from the model estimated clearance, median colistin concentration, and potency of colistin base can be compared with the actual dose to demonstrate applicability of model to actual dose received.

1 $$\text{Dose of colistin transited through the small intestine per day }\left(\mathrm{I}\text{U/kg BW/24 h}\right)= \frac{Clearance \left(\text{kg content/kg BW/h}\right)}{F}\times Median colistin concentration \left(\text{mg/kg}\right)\times 24h \times Potency of colistin base (\text{IU/mg})$$

With F = 100% for the gut compartment (there is no absorption).

Based on the median colistin concentration in the gut and the estimated clearance, the model estimated daily doses are 70,534, 91,627, and 139,935 IU/kg BW/day for the actually administered doses of 78,023 (nominal 75,000), 97,667 (nominal 100,000, and 141,133 (nominal 150,000) IU/kg BW/day respectively (Table 2).

**Table 2:** Model-estimated median daily colistin transit (as calculated from the median colistin concentration and clearance, Eq. (1) compared to the daily dose actually administered (back-calculated from water consumption on the day).

Targeted colistin dose (IU/kg BW/day)	Amount of colistin sulphate transit through the small intestine (mg/kg BW/day)	Amount of colistin base transit through the small intestine (IU/kg BW/day)	Mean daily dose of colistin administered (IU/kg BW/day)	Difference (%) between real and nominal dose
75,000	2.35	70,534	78,023	− 9.60
100,000	3.05	91,627	97,667	− 6.18
150,000	4.66	139,935	141,133	− 0.85

Only one random effect could be estimated due to the paucity of information. Between subject variability on the clearance parameter was 40.5% (eta shrinkage 48.5%) and as F = 0, this is a measurement of the variability of individual colistin intake from the computation of the daily amount transiting in the gut in Eq. (1). Goodness of fit plots for the compartment model are reported in Supplementary Figs. S1–S5.

### Monte-Carlo simulation (MCS)

**Figure 3:**
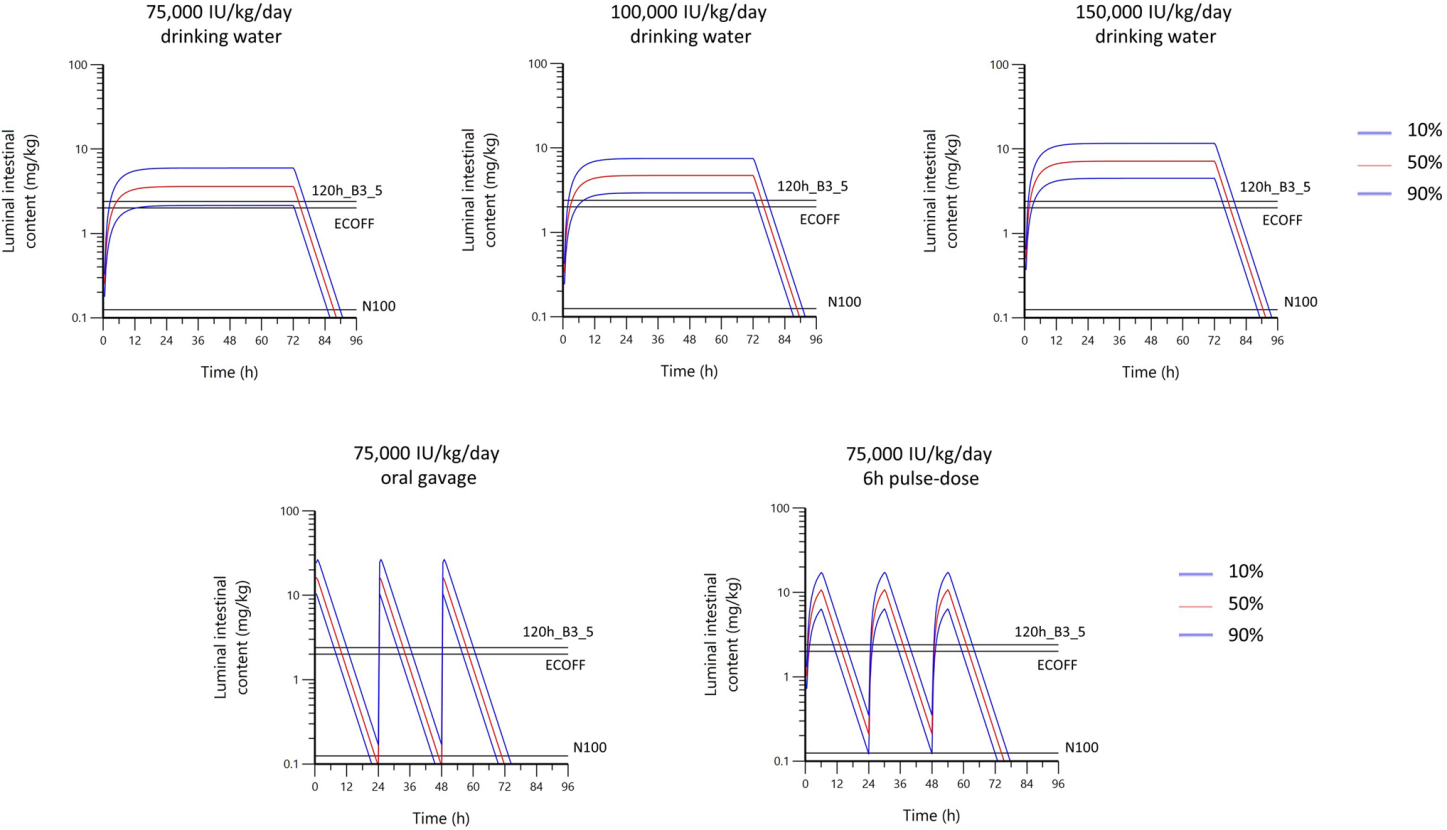
10th, 50th, and 90th percentile of the small intestine luminal colistin content along time, using a simple 1-compartment (physiologically naïve) model (simulation of 500 individuals), for dosage regimens of 75,000, 100,000, and 150,000 IU/kg/day in drinking water, 75,000 IU/kg oral gavage and 75,000 IU/kg/day as 6 h-pulse dose in water. Black horizontal lines represent ECOFF, MIC for *E. coli* N100 (*mcr*-negative) and *E. coli* 120h_B3_5 (*mcr*-positive) measured in CAMHB.

The 10^th^, 50^th^ and 90^th^ percentile of the small intestine luminal colistin content along time, using the simple 1-compartment model (simulation of 500 individuals), for all dosage regimens including a 6 h pulse-dose modality presenting a possible alternative to administration of the current clinical dose of 75,000 IU/kg BW/day, are displayed in Fig. 3 and Table 3, alongside MIC measured in cation-adjusted Mueller–Hinton broth (CAMHB).

**Table 3:**
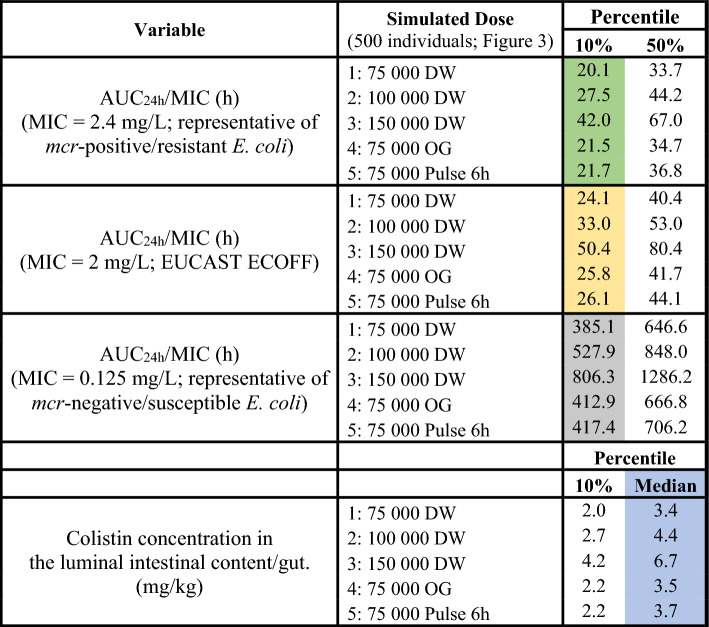
Computation of 10th and 50th percentile of AUC_24h_/MIC (h) for 3 MIC levels, using the 1-compartment classical (physiologically naïve) PK model.

Simulation was carried out for 5 dosage regimens. Percentiles; 10 and 50% (shaded column). MIC values in CAMHB were 2.4 mg/L for 120h_B3_5 (green), 2 mg/L for ECOFF (yellow) and 0.125 mg/L for N100 (grey). Median colistin concentration in luminal intestinal content (gut) expressed in mg colistin/kg intestinal content (blue).

#### In silico dose fractionation for determination of a PK/PD index for colistin using PK model

Dose fractionation was used to determine the optimal PK/PD indices and probability of target attainment (PTA) (Fig. 1). The simple 1-compartment PK model was used as input with a heteroresistant PD model, previously described by Mead et al.^26^ with the PD parameters for the representative *E. coli mcr*-negative strain N100 and *E. coli mcr*-positive strain 120h_B3_5 (reproduced in Supplementary Table S2), to compute the area under the bacterial curve AUC_bact(0–24 h)_, for the mixture and each of the sub-populations independently (S1 and S2). In brief, the prototype *mcr*-positive and *mcr*-negative bacteria shared most of the PD parameters except three of them, namely F1, F2 and Emax (see Supplementary Table S2). Therefore, two specific isolates that are *mcr*-negative would only differ by their EC_50_, scaling in line with their respective MIC. Same would apply to two *mcr*-positive isolates. This scaling was taken into consideration when extrapolating for isolates with an MIC at the ECOFF.

**Figure 4:**
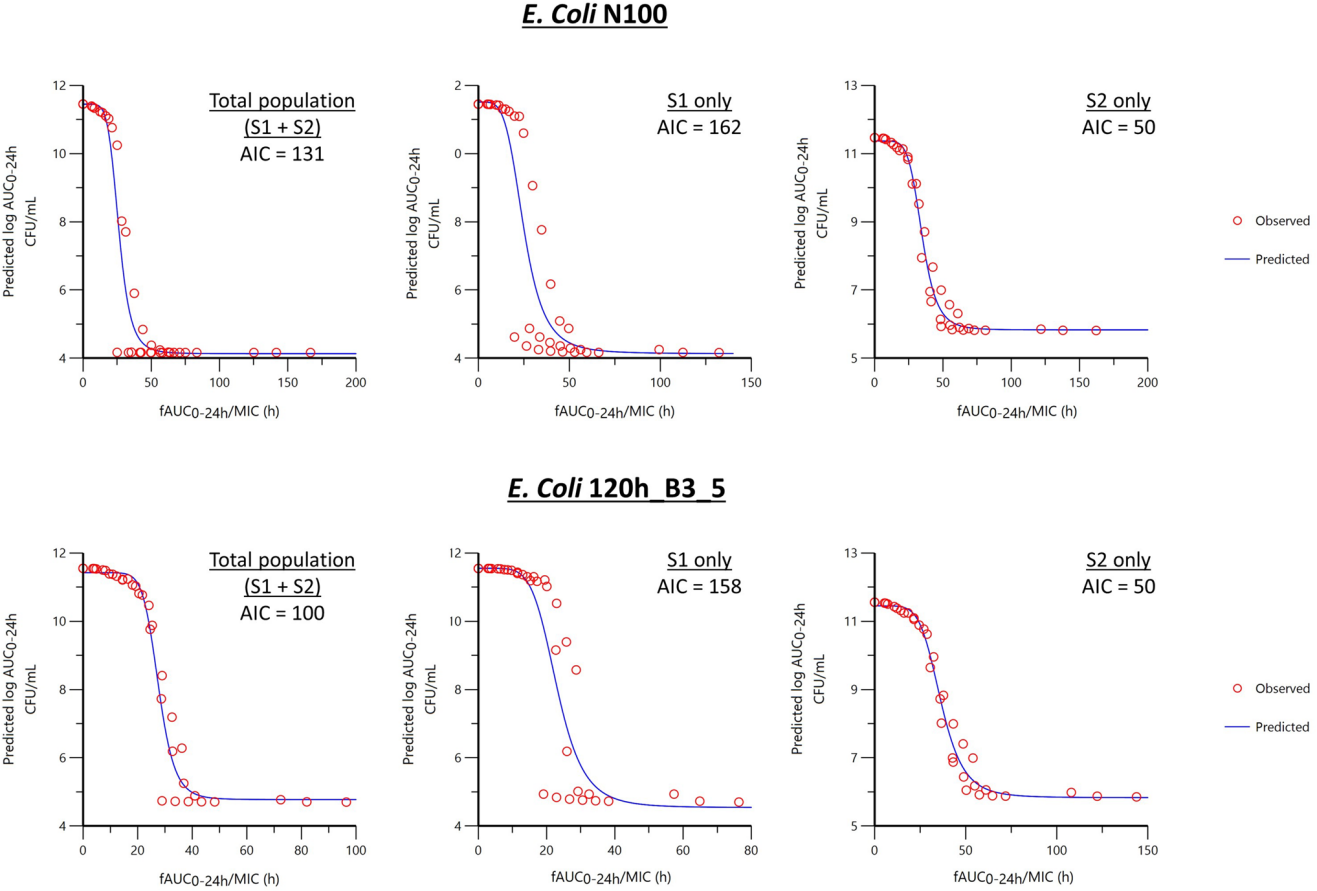
Semilogarithmic plot of the prediction of bacteriological effects estimated by the log 10 of the AUC of the bacterial population measured over 24 h i.e. AUC_bact(0–24 h)_ as a function of the selected PK/PD index *f*AUC_PK(0–24 h)_/MIC. In the absence of colistin, AUC_bact(0–24 h)_ is maximal and its log10 equal to 11.4 and a log10 of AUC_bact(0–24 h)_ equal or lower than 4 × indicates that colistin was able to eradicate the pathogen with cfu counts that fell below the limit of quantification of the analytic method i.e. 100 cfu/mL. Data were fitted with an I_max_ sigmoid model with *f*AUC_PK(0–24 h)_/MIC as a predictive variable and log_10_ AUC_bact(0–24 h)_ as dependent variable for *mcr*-negative isolate N100 and *mcr*-positive isolate 120h_B3_5 with fraction of free colistin at 0% protein binding.

Fitting comparison for prediction of log_10_ AUC_bact(0–24 h)_, via sigmoidal I_max_ model, versus either *f*AUC_(PK 0–24 h)_/MIC or *f*T > MIC (%) as the predictive variable indicated that *f*AUC_(PK 0–24 h)_/MIC as the optimal PK/PD index (Supplementary Fig. S5). *E. coli mcr*-negative representative strain N100 (MIC = 0.125 mg/L) at 0% protein binding had a better fit for *f*AUC_(PK 0–24 h)_/MIC (AIC = 131) than *f*T > MIC (AIC = 145). *E. coli mcr*-positive strain 120h_B3_5 (MIC = 2.4 mg/L) at 0% protein binding also had a better fit for *f*AUC_(PK 0–24 h)_/MIC (AIC = 100) than *f*T > MIC (AIC = 138). Figure 4 illustrates the fitting of the optimal PK/PD index against the total bacterial population (S1 + S2) and to each sub-population independently.

The critical value for the best fitting PK/PD index *f*AUC_(PK 0–24 h)_/MIC, seen in Table 4, to achieve 50% of the maximal in silico antimicrobial effect is 26.2 h for *mcr-*negative *E. coli* N100 for the total population. When the sub-populations are split to explore the critical value for either the S1 or S2 populations individually the critical values are 8.4 and 38.1 h respectively for S1 and S2, the heteroresistant subpopulation. This indicates that the average free colistin concentration over 24 h should be equal to 1.1-fold the (24 h measured) MIC, 0.35-fold the MIC and 1.59-fold the MIC for the total population, S1 sub-population and S2 sub-population respectively.

**Table 4:** Critical values of the PK/PD index *f*AUC_(PK 0–24 h)_/MIC (h) to achieve 50% of the maximal possible in silico effect for the combined population, for the S1 (susceptible) sub-population independently, and for S2 (less-susceptible) sub-population independently.

Representative strain	*f*AUC_(PK 0–24 h)_/MIC (h)*			Average free concentration (expressed as multiples of MIC, unitless) to achieve 50% kill effect		
	Total population (S1 + S2)	Sub-population S1 only	Sub-population S2 only	Total Heteroresistant population (S1 + S2)	Sub-population S1 only	Sub-population S2 only
*mcr*-negative *E. coli* N100 (MIC = 0.125 mg/L)	26.2	8.4	38.1	1.1	0.35	1.59
*mcr*-positive *E. coli* 120h_B3_5 (MIC = 2.4 mg/L)	27.6	18.7	37.9	1.2	0.78	1.58

Similarly, this critical value for the *mcr*-positive *E. coli* 120h_B3_5, was 27.6 h when considering the total population, but was 18.7 or 37.9 h when considering individual sub-populations, S1 and S2, respectively. This indicates that the average free colistin concentration over 24 h should be equal to 1.2-fold the MIC, 0.78-fold the MIC and 1.58-fold the MIC for the total population, S1 sub-population and S2 sub-population respectively.

#### Probability of target attainment

**Figure 5:**
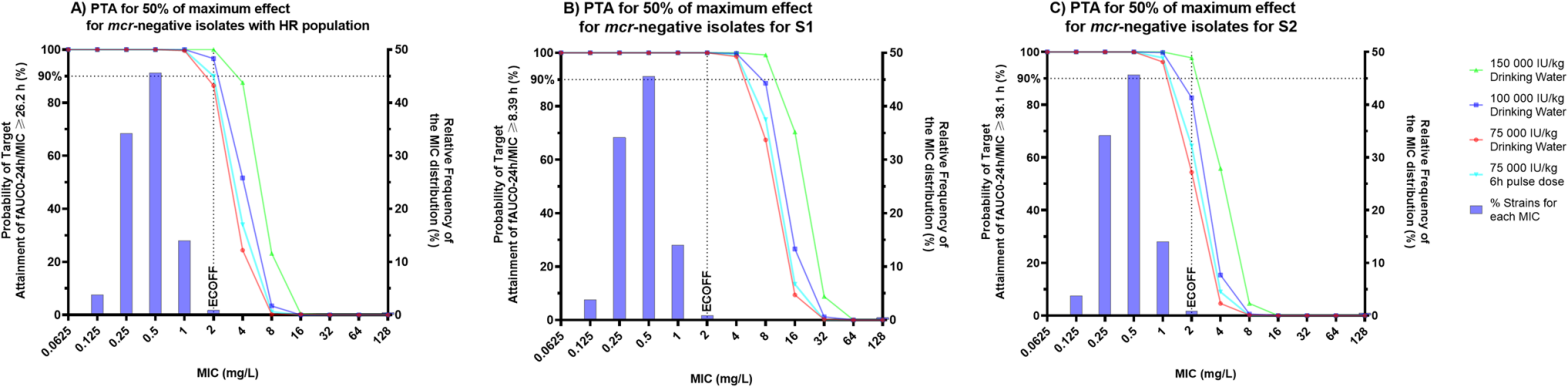
For *mcr*-negative *E. coli* probability of target attainment (PTA) for the pharmacodynamic targets (PDT) *f*AUC_(PK 0–24 h)_/MIC of: (**A**) ≥ 26.20 h for combined population, (**B**) ≥ 8.39 h for the highly susceptible dominant S1 (approx. 99.95% of the initial total population) population, or (**C**) ≥ 38.10 h for the less-susceptible sub-dominant S2 (approx. 0.05% of initial total population) population. PTA was determined in 500 simulated broiler chickens for four doses of colistin (75,000, 100,000, and 150,000 IU/kg via drinking water and 75,000 via 6 h pulse-dose drinking water). The horizontal dotted line signifies the PTA of 90%. The relative frequency of the MIC distribution of *E. coli* strains is taken from European Committee for Antimicrobial Susceptibility Testing (EUCAST, 2022).

**Figure 6:**
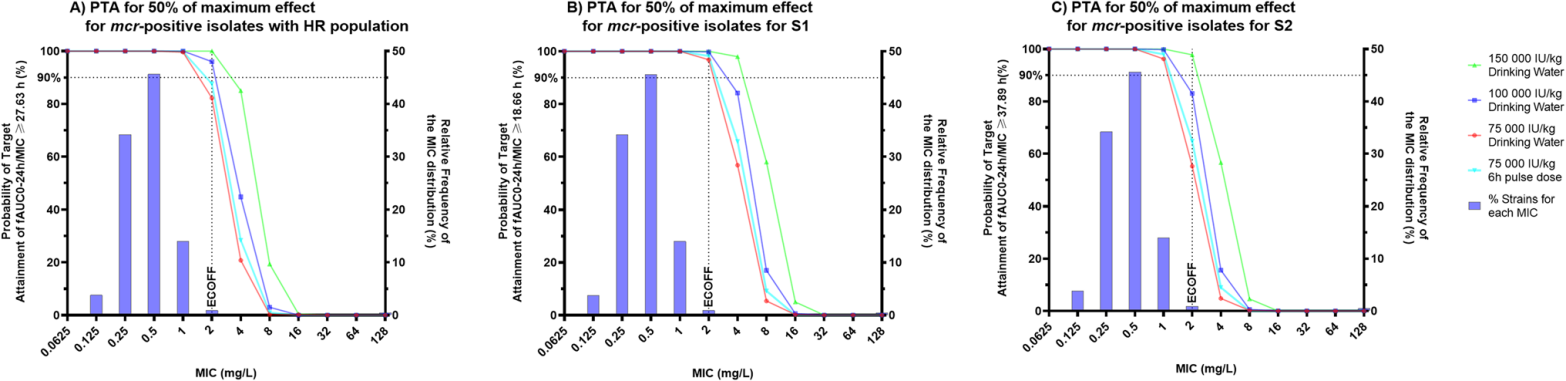
For *mcr*-positive *E. coli* probability of target attainment (PTA) for the pharmacodynamic targets (PDT) *f*AUC_(PK 0–24 h)_/MIC of: (**A**) ≥ 27.6 h for combined population, (**B**) ≥ 18.66 h for the highly susceptible dominant S1 (approx. 99.95% of the initial total population) population, and (**C**) ≥ 37.89 h for the less-susceptible sub-dominant S2 (approx. 0.05% of the initial total population) population. PTA was determined in 500 simulated broiler chickens for four doses of colistin (75,000, 100,000, and 150,000 IU/kg via drinking water and 75,000 via 6 h pulse-dose drinking water). The horizontal dotted line signifies the PTA of 90%. The relative frequency of the MIC distribution of *E. coli* strains is taken from European Committee for Antimicrobial Susceptibility Testing (EUCAST, 2022).

Assessment of the PK profiles for the simple 1-compartment model, considered as indicative of physiological models due to similar AUCs, generated using MCS were compared to the PD targets to achieve 50% of maximum bacterial kill. The results of the PTA analysis are displayed in Figs. 5 and 6.

For the total population (S1 and S2), this predicted that 90% of the population met the target for N100 (MIC = 0.125 mg/L) at all doses and for isolates with MIC up to 1 mg/L, considered the majority of wild-type *E. coli* based on the MIC distribution provided by EUCAST and with MIC_90_ based on distributions in Denmark, France, Netherlands and UK^27^. An increased dose of 100,000 IU/kg BW/day would achieve the target for putative HR bacterial population (S1 + S2) isolates with an MIC equal to the ECOFF (2 mg/L), although by using a 6 h pulse-dosing regimen still maintaining the current clinical dose of 75,000 IU/kg BW/day treatment of susceptible strains with MIC equal to ECOFF would be attainable in 90% of the population. However, for resistant isolates even with MIC close to the ECOFF, i.e. representative bacteria 120h_B3_5 (MIC = 2.4 mg/L), a further increase to 150,000 IU/kg BW/day would be required (Table 5).

**Table 5:**
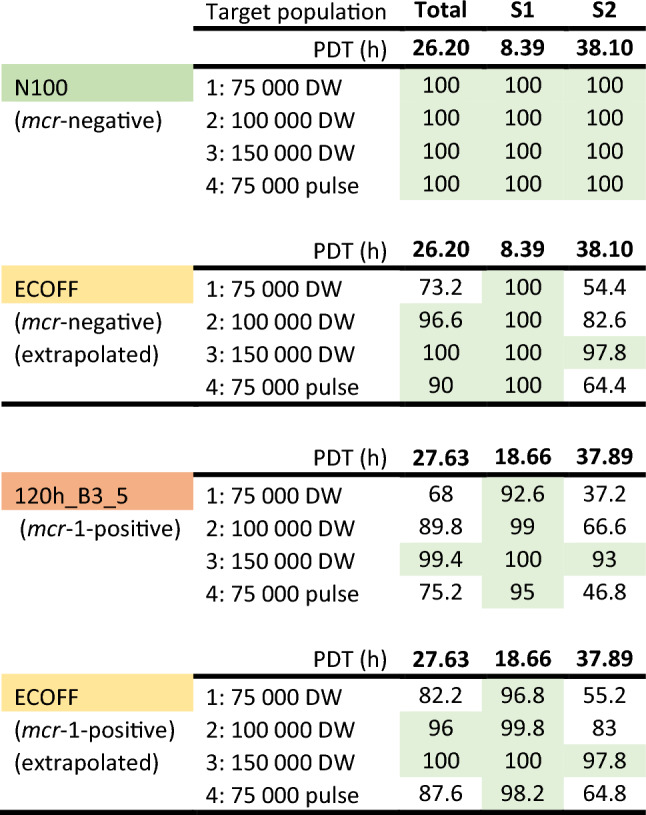
Summary table comparing the probability of target attainment (PTA%) for the estimated 50% pharmacodynamic AUC_24h_/MIC target values (PDT) for the total population, S1 sub-populations, and S2 sub-population for 2 representative bacteria (*mcr*-negative N100 and *mcr*-positive 120h_B3_5) having MIC of 0.125 and 2.4 mg/L respectively (see Table 4).

Results were obtained, by simulating the one-compartment model selected to describe the intestinal colistin disposition, with 4 dosage regimens (75,000, 100,000, 150,000 IU/kg/day 75,000 IU/kg/6 h pulse, in DW), in 500 individuals. For a bacterium with an MIC equal to ECOFF of 2 mg/L, both PD targets were explored (*mcr*-negative and positive) through extrapolation of the respective PD parameters. Green cells represent the attainment of the pharmacodynamic target in ≥ 90% of the individuals of the population.

Targeting the dominant susceptible sub-population S1, which is estimated as 99.95% of the initial population, it is anticipated that at MIC equal to the ECOFF we can still obtain a PTA of ≥ 90% for *mcr*-negative isolates with the clinical dose. For *mcr*-positive isolates at the ECOFF targeting the total population a slight increase of the dose to 100,000 IU/kg BW/day may be required but when targeting the S1 all simulated doses, including the current clinical dose, achieve ≥ 90% PTA.

## Discussion

Colistin is used in chickens for the treatment and metaphylaxis (following confirmation of disease within the flock) of susceptible (ECOFF = 2 mg/L) gastro-intestinal non-invasive *E. coli* infections. The dose recommended for administration via drinking water is 75,000 IU of colistin (as sulphate) per kg body weight per day for 3–5 consecutive days in Europe. As colistin is not absorbed from the gastrointestinal tract, its effect is exerted locally within the intestinal lumen and the wall of the intestine. As such, there is an opportunity to develop a PK model to predict the gut concentration throughout the day for a prolonged administration, after administration of different dosage regimen (typically over 3–5 days). In this study, a 3-day treatment period was retained in line with current trends to minimise antibiotic consumption through early treatment and optimised therapeutic regimen.

Various models have been proposed to predict AMD concentrations in the gut and circulation^4–6^, although it may be more accurate to model the physiological flow between gut segments taking into consideration digestive changes in line with feeding behaviour and circadian variation^7,8^. Optimally, this would involve measuring the AMD concentration in multiple gut segments across time, in line with the administered dose. Alternatives to repeated local sampling such as faecal sampling may be easily performed but may be flawed due to the mixture of faecal and urine material in birds. A more convenient option is destructive sampling to directly collect intestinal content to subsequently develop either a physiologically based model built on a priori knowledge of the gut flow and content around circadian cycle^18^ or a simple lumped compartmental model of the intestinal content. In this study, a simple 1-compartment model was used to predict the pharmacokinetics of colistin in the small intestine (jejunum and ileum). The estimated model parameters defined colistin transit (as IU/kg BW/day based on the clearance) and median intestinal colistin concentration within an acceptable range of the administered dose as back calculated based on water consumption. Differences up to 10% may be related to the assumption that all water loss was through consumption when dosed water may be lost from bell-drinkers through spillage, evaporation, and absorption from contaminating litter or related to a loss of colistin through binding to the bell drinker^28^. Aspects that should be investigated in future studies.

Through multidose fractionation (single, two or four fractions) performed in silico using the PD estimated model parameters of colistin effect combined with a basic PK function describing the colistin disposition as observed in chicken following oral gavage *f*AUC_(PK 0–24 h)_/MIC was selected as the optimal PK/PD index. This applied to both strains and when considering the sub-populations independently. Selection of *f*AUC_(PK 0–24 h)_/MIC as the best PK/PD index for colistin is consistent with previous reports in the literature for other organisms^29–32^ and for *E. coli*^33,34^. Critically, if the average concentration of colistin is maintained over 24 h at 1.1-fold the MIC and 1.2-fold the MIC for *mcr-*negative and *mcr*-positive strains respectively, a 50% maximal kill effect is achievable based on this PK/PD index (Table 4). Although it should be considered that colistin has been shown to have a very rapid, highly potent bactericidal effect on the predominant *E. coli* population even in the presence of heteroresistance^26^, that may indicate sufficiently high colistin concentrations reached even for largely shorter periods than 24 h (as considered for the *f*AUC) may have a significant impact. In fact, considering that the population is likely to be heterogenous with 99.95% of the population representing a highly-susceptible sub-population (S1; the less-susceptible sub-population S2 being only 0.05% of total population) we can posit that treatment aimed at this population may be sufficient to reduce the bacterial burden in the poultry intestine sufficiently to prevent enteric disease. In this case, to achieve an equivalent 50% maximal kill effect the average concentration of colistin maintained over 24 h would need to be only 0.35-fold the MIC and 0.78-fold the MIC for *mcr-*negative and *mcr*-positive strains respectively. It should be emphasized at this level that a 24 h measured MIC mainly reflects the MIC of the S2 subpopulation which showed growth after the rapid elimination of the dominant S1 population. Targeting the dominant S1 population would suggest a possible reduction of 32% of the daily dose from in vitro prediction, but this should be confirmed in new in vivo trials as we could not quantify *E. coli* in the present study.

Monte-Carlo simulations were used to identify the PTA for representative poultry isolates with varying MICs, including simulation of a possible 6 h-pulse dosing regimen that may increase colistin efficacy in vivo, in relation to its effect being partially concentration dependent (although full evaluation of maximum concentrations as PK/PD index was not performed). On this basis, the current clinical dose would achieve the critical PK/PD target in at least 90% of individual birds for susceptible *E. coli* with low MICs, based on the MIC as determined in CAMHB using standard in vitro methods. To achieve that coverage for higher MIC isolates (MIC = 2 mg/L), a higher dose is required or alternatively the use of a 6 h pulse-dose at 75,000 IU/kg/day would achieve 90% coverage when treating a target with MIC at 2 mg/L. However, at 1 dilution below the ECOFF (1 mg/L), PTA was > 95% for the registered dose for S2 *E. coli* populations, whether they harbour *mcr* or not. Under these conditions, a PK/PD cut-off at 1 mg/L would be 1 dilution lower than the ECOFF, validating 2 mg/L as a possible ECOFF for colistin in chicken. Extending this approach to investigate treatment of the two sub-populations represented by a heteroresistance model provides an alternative conclusion. For *mcr*-negative isolates, with MIC up to and including the ECOFF, the current clinical dose of 75,000 IU/kg BW/day via drinking water would be sufficient to achieve the pharmacodynamic target for treatment of the dominant S1 population, but not the minority S2 population. For *mcr*-positive isolates at the ECOFF, the current clinical dose falls marginally short of the 90% attainment target for the total population (82.2%) and it is predicted that a slight increase to 100,000 IU/kg BW/day would be required to exceed 90%. Alternatively, if we consider dosing to target the S1 population, maintaining the current dose may be sufficient to achieve a significant decrease in bacterial population across 96.8% of birds but adjusting the dosage regiment to a 6h pulse-dose regimen would increase this to achieve the target in 98.2% of birds. Overall, these predictions indicate that a shorter dosing period such as a 6h pulse-dose regimen increases probability of target attainment, but this would need to be validated in subsequent clinical trials. The lack of target attainment for the less-susceptible sub-population may provide an explanation for the apparent in vivo regrowth and selection of non-*mcr* resistance demonstrated previously by Mead et al.^3^. However, the immediate selection of the less-susceptible sub-population does not indicate true resistance but rather transient reduced susceptibility due to the unstable nature of monoclonal heteroresistance^35,36^. Regrowth of these populations long-term would be anticipated to show a similar population dynamic (i.e. revert back to F1 and 1-F1 proportions) and spectrum of susceptibility in line with the pre-treatment population.

In this situation treatment is used to lower the number of resident *E. coli* in the gastrointestinal tract and control gut overgrowth, in a similar fashion to selective decontamination^37,38^. As the outcome is not to achieve complete eradication (gut sterilisation), a lower effect than predicted by the PK/PD index critical value may be enough to reduce the bacterial burden within the gastrointestinal tract. In fact, a previous study in chickens has demonstrated that the current clinical dose of 75,000 IU/kg BW/day for 3-days reached maximum intestinal concentrations greater than 5 mg/kg, far beyond the MIC at ECOFF and of the *mcr*-positive strain indicated in this study, and that this resulted in an almost 1000-fold reduction in the *E. coli* population^3^.

When evaluating the degree of activity of colistin in vitro against poultry *E. coli* it is important to consider that all in vitro assessment is dependent on the experimental growth conditions. The use of artificial media, e.g. cation-adjusted Mueller–Hinton broth (CAMHB), may not be physiologically relevant^39^, and the MIC and time-kill analysis are markedly dependent on the growth medium^40,41^. Investigation of the pharmacodynamics of colistin in an intestinal growth medium have yet to be reported but may provide more physiologically relevant evaluation for dose estimation. Furthermore, antimicrobial binding to intestinal contents is likely to vary significantly depending on the specific composition of the gut contents, which varies greatly depending on location within the digestive tract^42,43^. To establish binding associated with the intestinal composition of the IGM, understanding that ultrafiltration and microdialysis method being compromised by non-specific binding, we propose to compare colistin loss following centrifugation of IGM (i.e. removal of the faecal/intestinal particulates) against CAMHB. We used a microbiological assay (not LCMS) to measure the remaining antimicrobial activity (colispot method using a *E. coli* ATCC 25922 bacterial lawn^44^). This resulted in 2% colistin activity loss from CAMHB and 27% loss activity from IGM (due to binding to particulate matter lost in centrifugation). This was a relatively low percentage difference and would result in colistin concentrations that are still high enough in intestinal media to be efficacious against the susceptible population. This apparent loss of in vitro activity is of the same order of magnitude than the margin on dose this study proposes, i.e. efficacy can still be achieved with a 32% reduction in dose. Critically, further studies are required to determine the unbound fraction of colistin in the small intestine (jejunum and ileum) to support the model assumptions and outcomes as determined in in vitro growth media. Target values of the PK/PD index were based on the growth achieved in in vitro conditions, whilst several works have indicated the bacterial growth and antibiotic activity are modified when in a natural bacterial community, such as the gut^45^, and may further impact on target attainment. Further improvements to this PK/PD approach should focus on implementing multi-compartmental sampling to measure the content across the entire gut, which would allow more accurate predictions beyond the small intestine, and to quantify binding of colistin in intestinal matrices.

## Conclusion

In conclusion, a simple 1-compartment pharmacokinetic model suitably predicts the mean retention time and colistin concentration within the poultry intestine. Integration of this PK model with a heteroresistant pharmacodynamic model coupled with dose fractionation indicates that fAUC_0–24 h_/MIC is the optimal PK/PD index for describing the action of colistin.

Determination of the pharmacodynamic target to achieve 50% of the maximal in vitro efficacy for the total S1 plus S2 population, indicates that the current clinical dose (75,000 IU/kg BW/day via drinking water) yields a PK/PD Cut-off of 1 mg/L (PTA ≥ 90% of individuals for the majority of colistin susceptible isolates within the wild-type distribution), but higher doses (e.g. 100,000 IU/kg/day) may be required to treat isolates with mixed HR populations at the ECOFF. Targeting the dominant susceptible population (S1), which may constitute 99.95% of the *E. coli* total population, indicates that the current clinical dose would be suitable for wild-type susceptible isolates. Furthermore, changing the administration regimen to a 6 h pulse-dose increases the probability of target attainment, and this may allow for the S1 population of *mcr*-positive isolates that have an MIC at the ECOFF of 2 mg/L to be targeted.

## Materials and methods

### Colistin

European Pharmacopoeia compliant Meiji Seika Pharma’s colistin sulphate (ColiMeiji®, hereafter ‘colistin’) consisting of a 78.53% mixture of colistin A (polymyxin E1) and colistin B (polymyxin E2) and was supplied by Wyjolab (Chaillac, France). Potency, as supplied (and reported by CoA), was 23,558 IU/mg colistin sulphate and was prepared immediately prior to dosing at a stock concentration of 2 × 10^6^ IU/mL colistin base adjusted based on the international standard potency of colistin base 30,000 IU/mg^44^.

### Animals and treatments

#### Ethical approval

This study was approved by the Royal Veterinary College ethics and welfare committee and carried out under ASPA (PPL: PCCBD6D98). All animal studies were performed according to the institutional guidelines and complied with the ARRIVE guidelines.

#### Animal husbandry

One hundred and seventy-five Ross 308 broiler chickens were group-housed and fed baby chick crumbs (Smallholder Range, Norfolk, UK), a feed free of coccidiostats and designed to feed from hatching to 6–8 weeks and given free access to water for the duration of the study. Birds were housed in a 25-m^2^ floor pen with access to 5 × 3-L drinking bells (Farm & Country, Lincolnshire, UK), refreshed every 24 h. Average relative humidity was 44.5% (range: 29–63%) and average temperature 24 °C (range: 20–28 °C).

#### Colistin administration

Pharmacokinetic data from in vivo clinical and supra-clinical doses via drinking water and oral gavage were collected as follows. Birds were provided a 14-h photoperiod aligned with onset of dosing (0 h) at 07:00. Birds were housed together upon arrival and separated at 13-days into the following dosing groups 75,000 IU/kg oral gavage (n = 17), 150,000 IU/kg oral gavage (n = 17), 75,000 IU/kg/day drinking water (n = 38), 100,000 IU/kg/day drinking water (n = 38), and 150,000 IU/kg/day drinking water (n = 38), the remaining birds constituted an untreated control group (n = 27) housed in another room. Colistin was administered as a single oral gavage via manual syringe directly into the crop, with the volume calculated based on bird weight immediately prior to dosing. For colistin administration via drinking water, dose was calculated based on average bird weight and water consumption measured the preceding day. It was refreshed every 24 h over the 3-day dosing. The “real” dose received every 24 h was back calculated based on measured water consumption for each dosing period.

#### Collection of luminal intestinal content and determination of colistin content

For administration via drinking water, 4 birds were sampled at each of the following timepoints pre-dose (0 h), during-dose (12, 36 and 54 h) and post-dose (72, 75, 76, 78, 84 and 96 h). Following oral gavage, 2 birds were sampled at each of the following timepoints 0 h (pre-dose), 1 h, 3 h, 6 h, 12 h, and 24 h (post-dose). Birds were sacrificed through cervical dislocation. The whole small intestine was excised from the duodenum to the large intestine, including the caeca, using individually wrapped and sterilised sets of dissection apparatus were used to avoid cross-contamination between birds. The jejunum and ileum were resected (between proximal ligature after duodenum, and distal ligature before the large intestine) and luminal intestinal content (LIC) was collected as an homogenous sample extracted from the small intestine via peristaltic massage in a proximal-to-distal direction as previously described^3^, with the jejunum/ileum representing the principle compartment for potentially pathogenic *E. coli* (as compared to the large intestine). All samples were stored at − 80 °C prior to further analysis for no-more than 3 months. Following a previously validated method^45^, colistin was extracted from the matrix using solid-phase extraction with a methanol:water (1:2; v/v) solution. Polymyxin B1 was used as an internal standard. Colistin A and colistin B, (main components of colistin), were separated, detected and measured using ultra-high-performance liquid chromatography coupled with tandem mass spectrometry (UHPLC-MS/MS). Total colistin was calculated as the ratio of the sum of peak areas of colistin A and B over the polymyxin B1 peak area. With limit-of-quantification of 1.1 mg/kg and inter- and intra-day precision less than 13.3% and 15% respectively based on VICH GL49 guidelines, although linearity was demonstrated down to 0.55 mg/kg considered here as the limit of detection (LOD) and is in line with the mode of wild-type distribution for *E. coli* MIC^45^.

### Pharmacokinetic modelling

Colistin (converted in mg/kg BW) was deposited in a dosing compartment (crop), either as a single bolus input representing an oral-gavage administration or as a daily zero-order input lasting 24 h representing a continuous dose via drinking water. The colistin content pooled jejunum and ileum content (expressed as mg colistin/kg intestinal content) was assumed to be well mixed and was fitted with a one-compartment model. The model was parametrised with 3 parameters: “clearance” that is here an intestinal flow rate (expressed in kg intestinal content/kg BW h^−1^), “volume of distribution”, expressed in kg intestinal content/kg BW) and absorption rate constant for extravascular administration (Ka expressed in h^−1^).

Despite transit time of few hours during daytime. The content of the small intestine is never empty and fluctuates between approx. 2.2 and 0.50% of BW during day night cycles for birds of 27 days exposed to 14/10 h light cycle.^18^. A physiologically-based “gut” model, building on a priori knowledge of the gut flow and content around circadian cycle was initially used to assess the transit of colistin through the gastrointestinal tract of the chicken, specifically in the small intestine. This model led to almost the same conclusions and recommendations as the simple homogenous compartmental model and is not presented.

Between subject variability (BSV) was applied to the clearance (Cl) described using an exponential model, as in the following general equation:

$${P}_{i} = tvP * exp(\eta {P}_{i})$$

where tvP is the typical value of the parameter within the population and the random parameter ηP_i_ (*eta*) represents the deviation from the typical value for the ith individual*. Etas* are assumed normally distributed with a mean of 0 and a variance of ω^2^.

Between subject variability (BSV) was expressed as coefficients of variation with the following equation:

$$\mathrm{CV}\left(\mathrm{\%}\right)=100\times \sqrt{\mathrm{exp}\left({\upomega }^{2}\right)-1}$$

For model evaluation, a significant decrease (> 6.635 corresponding to p < 0.01 for one degree of freedom) in the Bayesian information criterion (BIC) as well as observation of observed vs. population, individual predicted concentrations plots, and visual predictive checks (VPC) were utilized. All concentrations lower than the LOQ of the bioanalytical method (1.1 mg/kg) were considered as censored and managed with the M3 approach and using the Laplace engine by Phoenix™ v8.3.0.5005 (Certara, St. Louis, Missouri). Residual variability was described with a multiplicative error model, providing optimal fit compared to additive or additive/multiplicative models. Residual variability was low due to the single random effect on the clearance considering only a single-point for each bird (due to the nature of the destructive sampling) and the near normal distribution of the Etas. The final population model was evaluated using a Visual Predictive Check (VPC; n = 300) that compares observed with predicted quantiles (10th, 50th and 90th percentiles).

### Monte Carlo simulation

Monte Carlo simulation (n = 500 birds per dosage regimen) were performed with the final model, for each of the following dosage regimens (i) Standard dose (75,000 IU/kg/day in drinking water), (ii) intermediate dose (100,000 IU/kg/day in drinking water), (iii) high dose (150,000 IU/kg/day in drinking water), (iv) Oral gavage (75,000 IU/kg/day as a single bolus), and pulse dosing (75,000 IU/kg/day in drinking water, administered over the first 6 h of the day). With pulse-dosing considered as an alternative style of administration regimen that may increase efficacy while retaining the current clinical daily dose.

### In silico dose fractionation for determination of a PK/PD index for colistin

Selection of the optimal PK/PD index for colistin was calculated using the in-silico PK/PD model dose fractionation. A previously validated PD model (from TCK analysis in CAMHB, Mead, et al.^26^) was combined with a hypothetical 1-compartment extravascular administration PK model, to simulate predicted colistin concentration, C(t). This PK model used an elimination half-life estimated through disappearance of colistin from the small intestine following dosing via oral gavage (set to 1 h). Using the PK/PD in silico model, dose fractionation was conducted for a range of doses as a single administration, two administrations at 12 h intervals, or 4 administrations at 6 h intervals, yielding a total of 36 possible exposure patterns. Simulations were performed with an initial bacterial load of 10^5^ CFU/mL, for representative *mcr*-negative (farm isolate N100; MIC = 0.125 mg/L) and *mcr*-1-positive (clinical isolate 120h_B3_5; MIC = 2.4 mg/L) *E. coli*, and for putative *mcr*-negative and *mcr*-positive bacteria with an MIC at the ECOFF (MIC = 2 mg/L).

For the bacteriological response, the cumulative area under the curve of the total bacterial count over 24 h (AUC_bact24h_) was used. Data were then log_10_ transformed for regression. As LOQ was set at 100 CFU/mL, it was considered that re-growth would not occur if the total bacterial population decreased to this value. PK/PD indices are conventionally determined using protein unbound (free; *f*) concentration. Bergen et al.^46^ indicated practical equivalence of total and unbound colistin in CAMHB (unbound fractions of 0.96 and 0.95 at 10 and 30 mg/L respectively). Furthermore, as colistin binding cannot be determined by ultrafiltration due to membrane adsorption, to establish binding associated with the intestinal composition of the IGM, we explored colistin loss following centrifugation of IGM (i.e. removal of the faecal/intestinal particulates) against CAMHB. Note that we used a microbiological assay (not LCMS) to measure the remaining antimicrobial activity (colispot method using a *E. coli* ATCC 25922 bacterial lawn). This resulted in 2% colistin activity loss from CAMHB and 27% loss activity from IGM (due to binding to particulate matter lost in centrifugation). This is a relatively small difference and as colistin exhibits reversible binding and activity when bound it was considered concentrations in the intestine would still reach significant concentrations. Subsequently, simulations were performed with amount of bound colistin at 0%. The area under the concentration–time curve over the MIC (*f*AUC_PK(0–24 h)_/MIC) and percentage of time the concentration exceeded MIC within 24 h (*f*T > MIC%) were computed, and fitted versus the AUC_bact24h_ for each strain with an Inhibitory Effect Sigmoid I_max_ PD model (Phoenix Model 108):

$$Effect={E}_{0}- \left(\frac{{I}_{max}\times {Index}^{\gamma }}{{Index}_{50}^{\gamma }+ {Index}^{\gamma }}\right)$$

where E_0_ is the maximum effect (obtained AUC_bacteria_ for the control curve for *C*(*t*) = 0), the maximum possible observed effect is (E_0_-I_max_), I_max_ being the amplitude of maximal effect. *Index*_*50*_ is the magnitude of the index (*f*AUC_PK(0–24 h)_/MIC or *f*T > MIC%) that achieves 50% of the I_max_, and *γ* is the sigmoidicity factor reflecting the steepness of the relationship. The coefficients of determination (*R*^2^) and visual inspection of graphs were used to select the PK/PD index that best predicted the antibacterial effect.

### Probability of target attainment

The PK/PD_CO_ is the highest MIC for which 90% of the simulated population achieves the PK/PD target^24^. Individual predicted intestinal colistin AUC_0-72 h_ was divided by 3 to yield AUC_24h_, from which the average predicted intestinal colistin content in the small intestine was computed. AUC_24h_/MIC was computed for a range of MICs corresponding to the EUCAST distribution of MICs for *E. coli* including 3 specific MICs: 0.125 mg/L which is the MIC for N100 (*mcr*-negative), 2 mg/mL for ECOFF and 2.4 mg/L which is the MIC for 120h_B3_5 (*mcr*-positive), all measured in CAMHB. The 90th percentile of the distribution AUC_24h_/MIC, computed for each dosage regimen with the model, were compared with the pharmacodynamic target values.

### Supplementary Information

Supplementary Information.

## Data Availability

The datasets generated for this study are available on request to the corresponding author.
