# Supplementary material for: Targeted dosing for susceptible heteroresistant subpopulations may improve rational dosage regimen prediction for colistin in broiler chickens
Source: Sci Rep. 2023 Aug 7;13:12822. doi: 10.1038/s41598-023-39727-w (PMC10406827; doi:10.1038/s41598-023-39727-w)
Supplement: Supplementary file 1 — Supplementary Information. [file 41598_2023_39727_MOESM1_ESM.docx]

Supplementary material

Supplementary Table 1: Back-calculated dose for chickens administered colistin via drinking water at 75 000 IU/kg/day drinking water (n = 38), 100 000 IU/kg/day drinking water (n = 38), and 150 000 IU/kg/day drinking water (n = 38).

| **Day** | **Water volume drunk per bird over 24h (mL)** | **Average weight of the sample of chicken (g)** | **Volume of (2,000,000 IU/mL) stock solution added to 1L** | **Actual dose for the day (IU/kg/d)** | **Actual dose for the day (mg/kg/d)** |
| --- | --- | --- | --- | --- | --- |
| **75 000 IU/kg/day** | | | | | |
| **Day 1**  **(0 – 24 h)** | 30.74 | 163.7 | 0.188 mL | 70,698 | 2.36 |
| **Day 2**  **(24 – 48 h)** | 40.61 | 171.7 | 0.179 mL | 84,545 | 2.82 |
| **Day 3**  **(48 – 72 h)** | 43.97 | 156.0 | 0.140 mL | 78,824 | 2.63 |
| **Mean (0 – 72 h)** | | | | **78,023** | **2.60** |
| **Total (0 – 72 h)** | | | | **234,068** | **7.81** |
| **100 000 IU/kg/day** | | | | | |
| **Day 1**  **(0 – 24 h)** | 26.21 | 156.3 | 0.239 mL | 80,264 | 2.68 |
| **Day 2**  **(24 – 48 h)** | 37.75 | 176.0 | 0.282 mL | 121,173 | 4.04 |
| **Day 3**  **(48 – 72 h)** | 42.3 | 179.6 | 0.194 mL | 91,563 | 3.05 |
| **Mean (0 – 72 h)** | | | | **97,667** | **3.26** |
| **Total (0 – 72 h)** | | | | **293000** | **9.77** |
| **150 000 IU/kg/day** | | | | | |
| **Day 1**  **(0 – 24 h)** | 31.00 | 157.0 | 0.333 mL | 131,367 | 4.38 |
| **Day 2**  **(24 – 48 h)** | 33.24 | 162.4 | 0.340 mL | 139,104 | 4.64 |
| **Day 3**  **(48 – 72 h)** | 40.7 | 171.5 | 0.323 mL | 153,511 | 5.12 |
| **Mean (0 – 72 h)** | | | | **141,133** | **4.70** |
| **Total (0 – 72 h)** | | | | **423,981** | **14.13** |

**Supplementary Table 2**: Typical value (tv) and median parameter estimates and 95% confidence intervals of the semi-mechanistic model.

| **Parameters^1^** | **Unit** | **Typical value (tv) estimate** | **Median (Bootstrap)**  **estimate** | **Confidence interval**  **(from Bootstrap)** | |
| --- | --- | --- | --- | --- | --- |
|  |  |  |  | **2.5% CI** | **97.5% CI** |
| **Bacterial growth system parameters** | | | | | |
| K_growthmax_ | h^-1^ | 2.44 | 2.39 | 2.25 | 3.66 |
| K_death_ | h^-1^ | 0.179 (fixed) | - | - | - |
| B_max_ | CFU/mL | 1.43 x 10^10^ | 1.49 x 10^10^ | 8.77 x 10^9^ | 1.98 x 10^10^ |
| alphaS1 | h^-1^ | 0.88 | 0.99 | 0.38 | 1.29 |
| alphaS2 | h^-1^ | 3.74 | 3.43 | 3.04 | 4.34 |
| F_1_ *mcr*-negative  (number of bacteria in S_1_ vs. S_2_) | - | 0.9995  (499 731 vs 269; 0.05%) | 0.9996  (499 760 vs 240; 0.05%) | 0.9992 | 0.9997 |
| F_1_ *mcr*-positive  (number of bacteria in S_1_ vs. S_2_) | - | 0.9994  (499 637 vs 363; 0.07%) | 0.9994  (499 692 vs 308; 0.06%) | 0.9989 | 0.9997 |
| **Colistin pharmacodynamic parameters** | | | | | |
| EC_50__S1 (*E. coli* N100; *mcr*-negative) | mg/L | 0.083 | 0.085 | 0.047 | 0.099 |
| EC_50__S2 (*E. coli* N100; *mcr*-negative) | mg/L | 0.115 | 0.115 | 0.106 | 0.158 |
| EC_50__S1 (*E. coli* 120h_B3_5; *mcr*-1) | mg/L | 2.760 | 2.624 | 2.340 | 3.408 |
| EC_50__S2 (*E. coli* 120h_B3_5; *mcr*-1) | mg/L | 2.174 | 2.063 | 1.907 | 3.634 |
| gamma_S1 | scalar | 4.09 | 3.91 | 3.37 | 8.84 |
| gamma_S2 | scalar | 3.22 | 3.80 | 1.79 | 8.38 |
| F_2_ (for *mcr*-negative isolates) | scalar | 12.66 | 13.65 | 5.93 | 16.61 |
| F_2_ (for *mcr*-positive isolates) | scalar | 3.76* | 3.93* | 1.72 | 6.13 |
| E_max_ (S2) for both *mcr*-negative and *mcr*-positive isolates | h^-1^ | 2.69 | 2.51 | 2.04 | 5.29 |
| E_max_ (S1) negative *mcr* isolates | h^-1^ | 34.12** | 34.44** | 28.61 | 38.44 |
| E_max_ (S1) *mcr*-positive isolates | h^-1^ | 10.13** | 10.43** | 7.58 | 13.66 |
| ^1^ *K_growthmax_, maximal growth rate constant; K_death_, natural death rate; B_max_, maximum possible bacterial density; alpha, delay before reaching maximal growth rate; F_1_, proportion of starting inoculum in subpopulation S1; EC_50,_ concentration required to achieve 50% of E_max_;* γ_S1*, Hill coefficient for sub-population S1;* γ*_S2, Hill coefficient for sub-population S2; F_2_, potentiation factor for K_drug_ on subpopulation S1; F_2MCR1_, potentiation factor for K_drug_ on subpopulation S1 for mcr-positive strains; E_max_, maximal increase in kill effect in addition to K_death_.*  * F_2MCR_ calculated from covariate parameter (dF_2MCR_) as F_2MCR_ = F_2_ x exp(dF_2MCR1_)  ** E_max_ (S1) = E_max_ (S2) x F_2_ | | | | | |


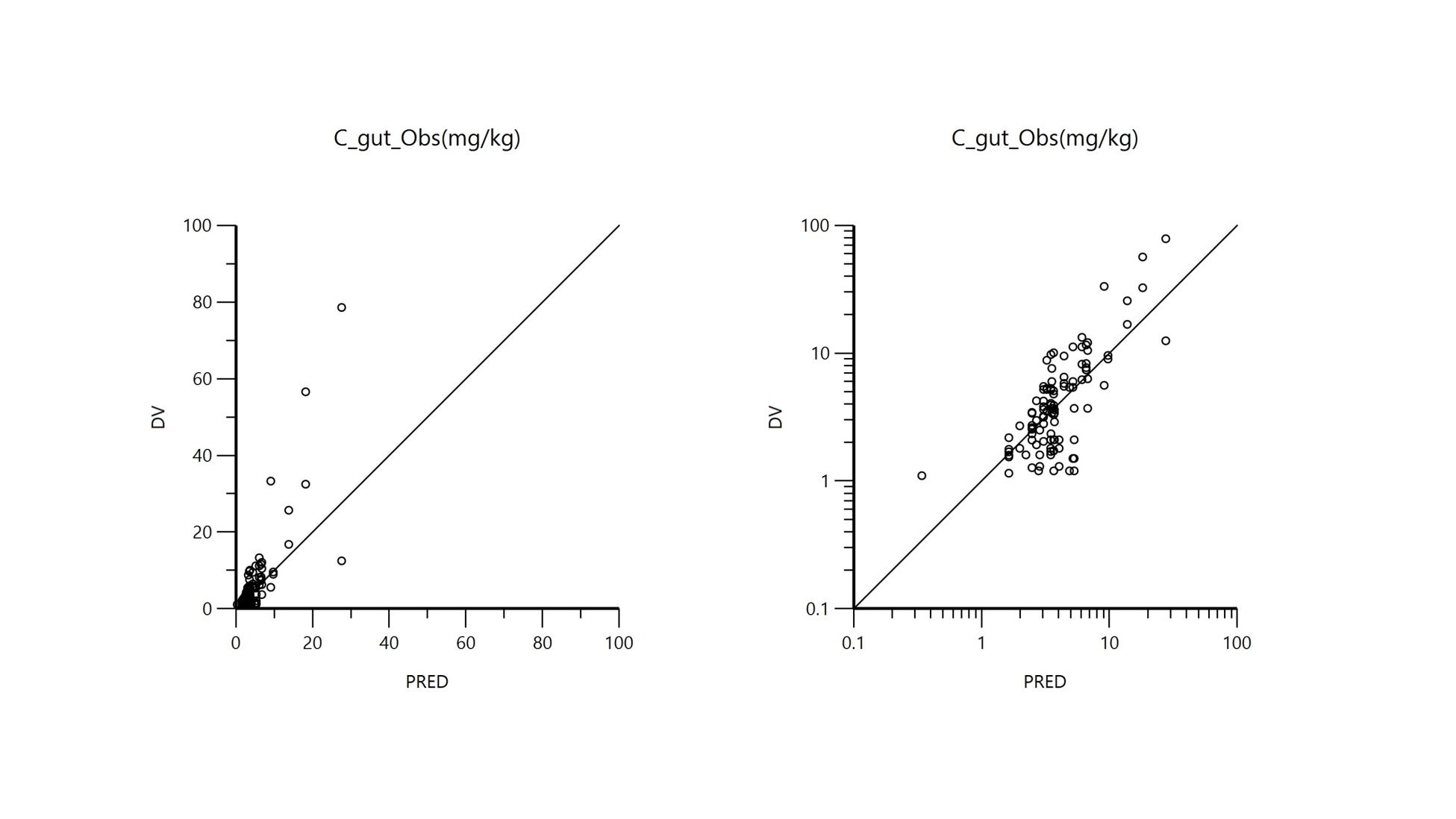


Supplementary Figure 1: DV vs PRED. Plot of the dependent variable i.e. of observed LIC colistin total concentrations (mg/L) versus predicted LIC colistin total concentrations (PRED). Prediction is obtained by setting random effects to the 'post hoc' or empirical Bayesian estimate of the random effects for the individual from which the plasma concentration observation was made. Thus, the plot shows observed vs fitted values of the model function. Ideally, they should fall close to the line of unity y=x. Arithmetic scale (left) and logarithmic scale (right).


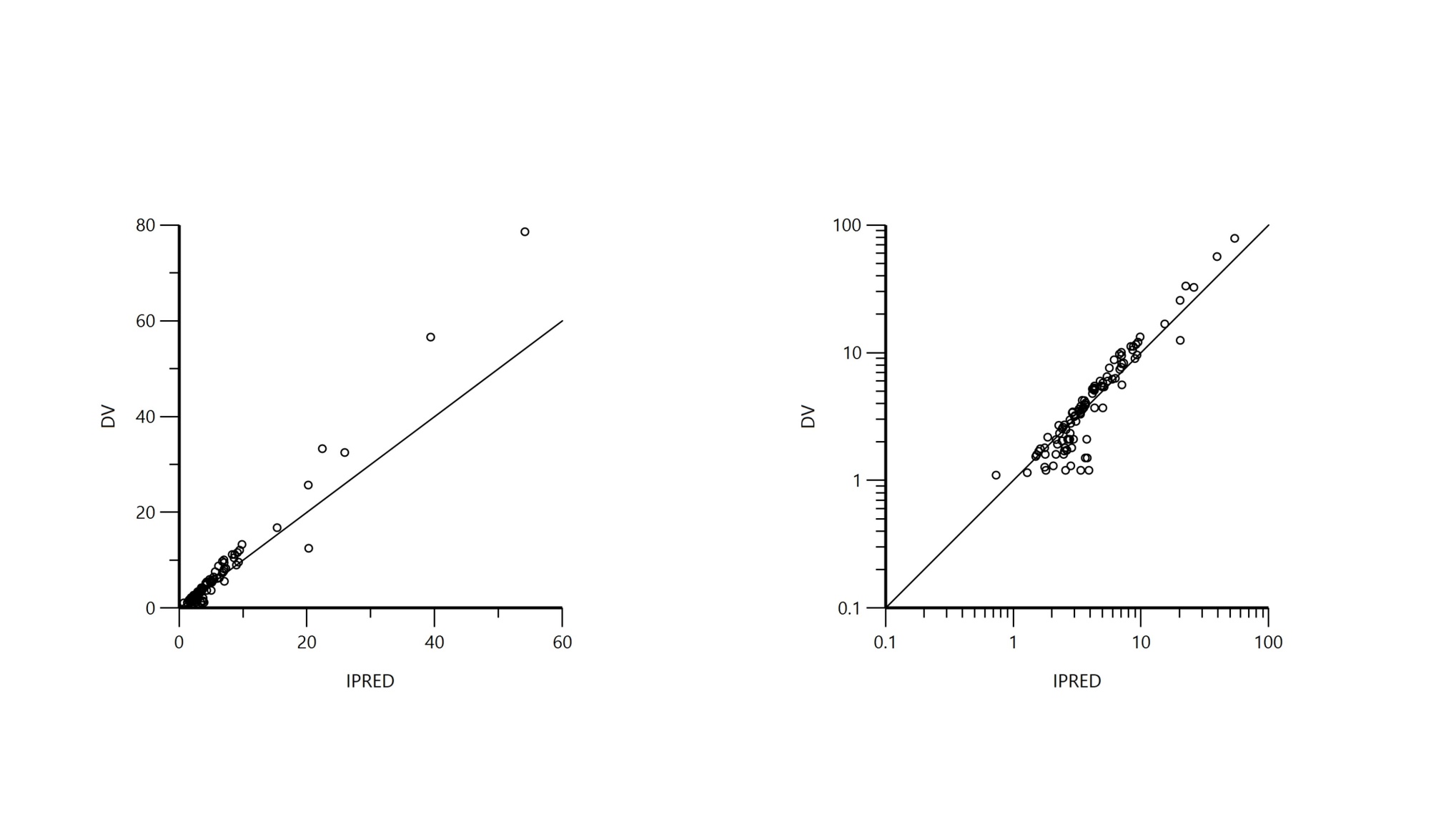


Supplementary Figure 2: DV vs IPRED. Plot of the dependent variable i.e. of observed LIC colistin total concentrations (mg/L) versus predicted LIC colistin total concentration for each individual (IPRED). Individual prediction is obtained by setting random effects to the 'post hoc' or empirical Bayesian estimate of the random effects for the individual from which the plasma concentration observation was made. Thus, the plot shows observed vs fitted values of the model function. Ideally, they should fall close to the line of unity y=x. Arithmetic scale (left) and logarithmic scale (right).


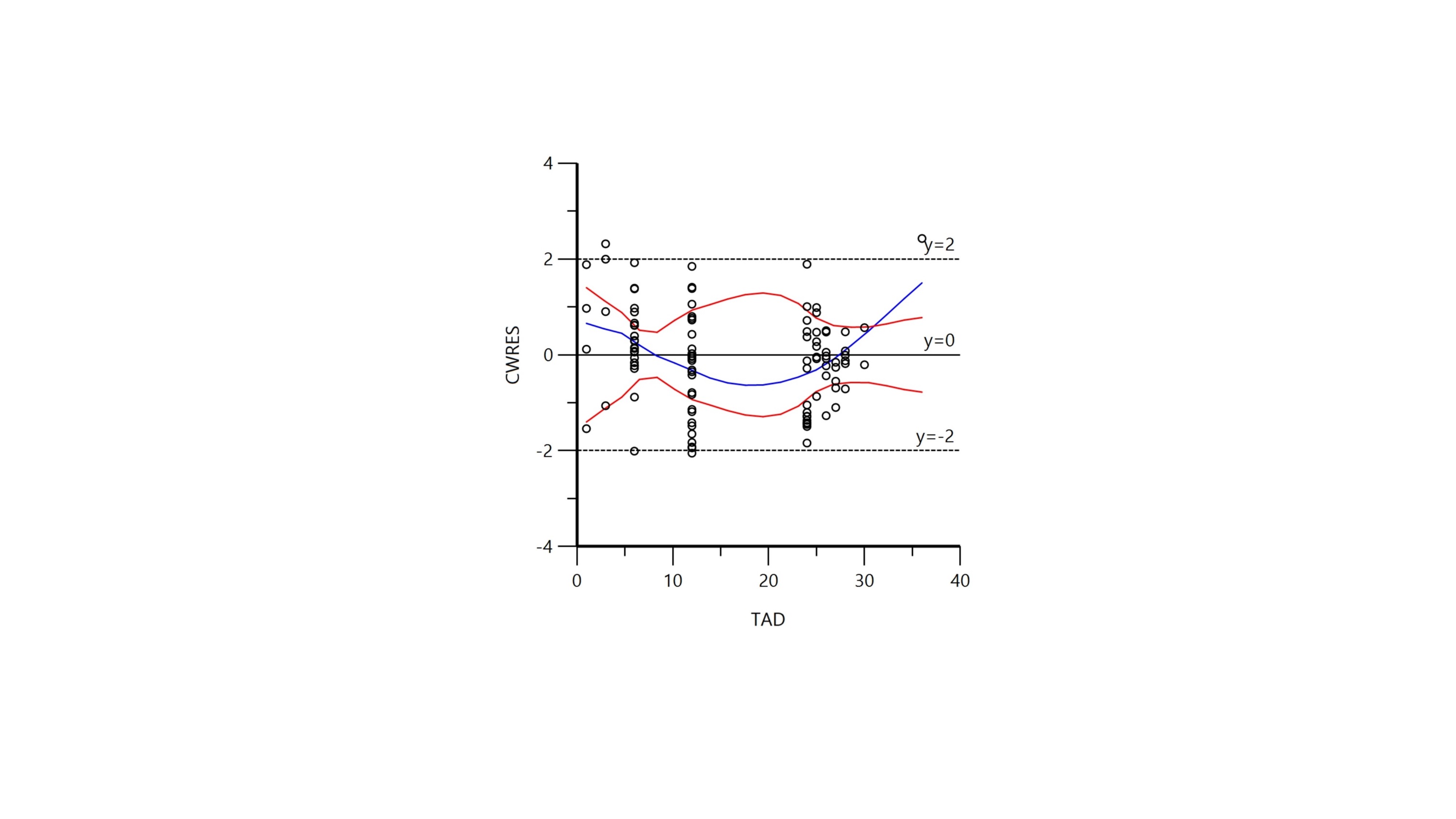


# Supplementary Figure 2: CWRES vs Time after administration: Plot of CWRES (conditional weighted residuals), a proposed replacement for the classical WRES (weighted residuals) goodness of fit statistic, against IVAR (time). Values of CWRES should be approximately N(0,1) and hence concentrated between y=-2 and y=+2. Values significantly above 3 or below -3 are suspect and may indicate a lack of fit and/or model misspecification. Red and blue curves are LOcally wEighted Scatterplot Smoothing (LOESS) regression curves. The blue curve takes into account the sign of the residuals (positive or negative), while the red curve and its reflection only consider absolute values of residuals. Ideally, the blue line should be at 0, and the red line (with its negative reflection) should not show any fanning.


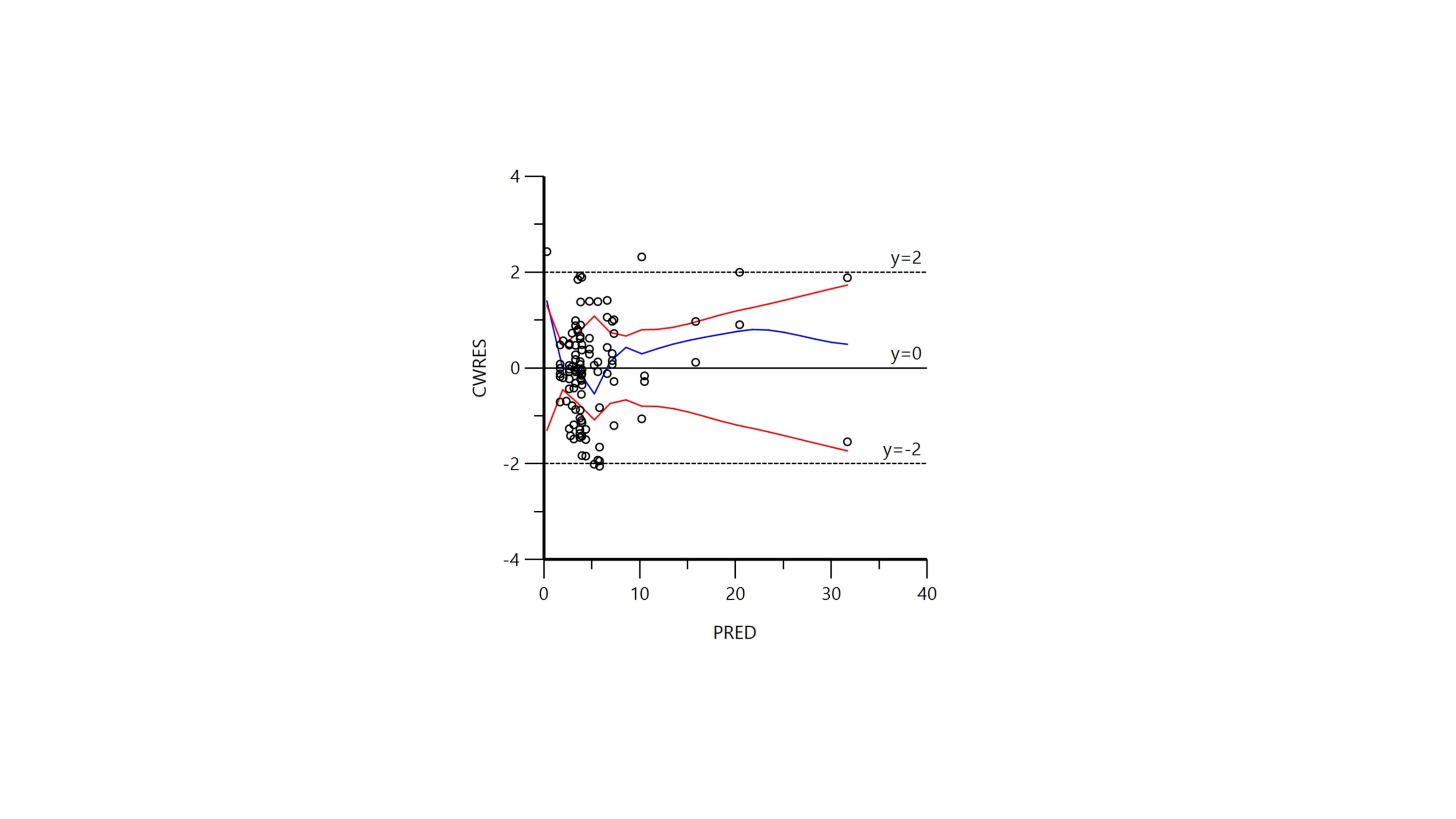


Supplementary Figure 3: CWRES vs PRED: Plot of CWRES (conditional weighted residuals), a proposed replacement for the classical WRES (weighted residuals) goodness of fit statistic, against PRED i.e. the population predictions used for the x axis. Values of CWRES should be approximately N(0,1) and hence concentrated between y=-2 and y=+2. Values significantly above 3 or below -3 are suspect and may indicate a lack of fit and/or model misspecification. Red and blue curves are LOcally wEighted Scatterplot Smoothing (LOESS) regression curves. The blue curve takes into account the sign of the residuals (positive or negative), while the red curve and its reflection only consider absolute values of residuals. Ideally, the blue line should be at 0, and the red line (with its negative reflection) should not show any fanning.


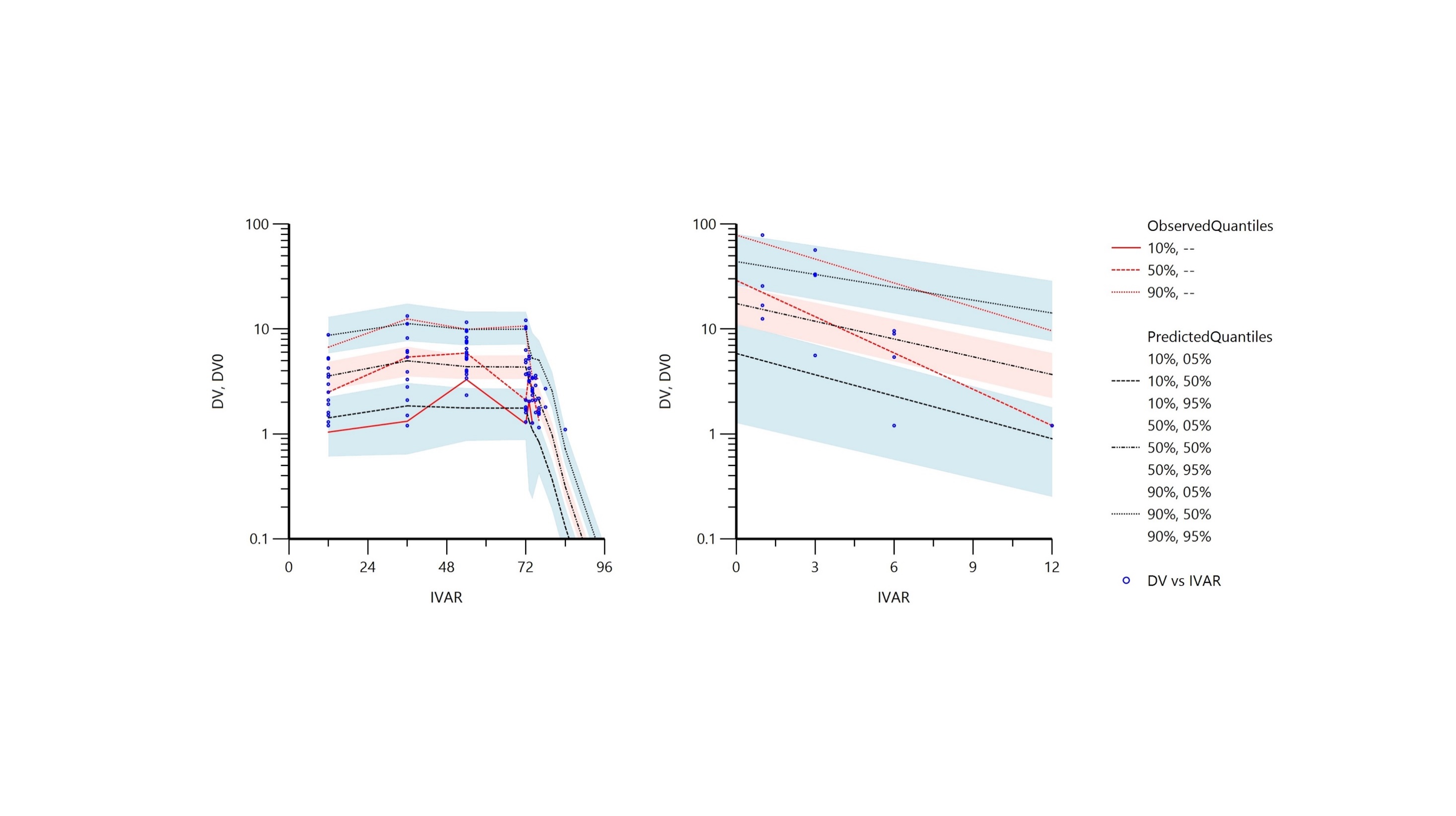


Supplementary Figure 4: VPC: Visual Predictive Check (VPC) for the drinking water (left) and gavage (right) administration obtained with 100 replicates of the dataset. For each stratification, the observed quantiles (10, 50 and 90%) are rather well superimposed with the corresponding predictive check quantiles over the observed data. Theoretically about 20% of data should be outside the plotted quantiles. Red lines: observed quantiles; Black lines: predicted quantiles; Black symbols: observed data. With the 90% confidence interval of predicted quantiles (shaded area).


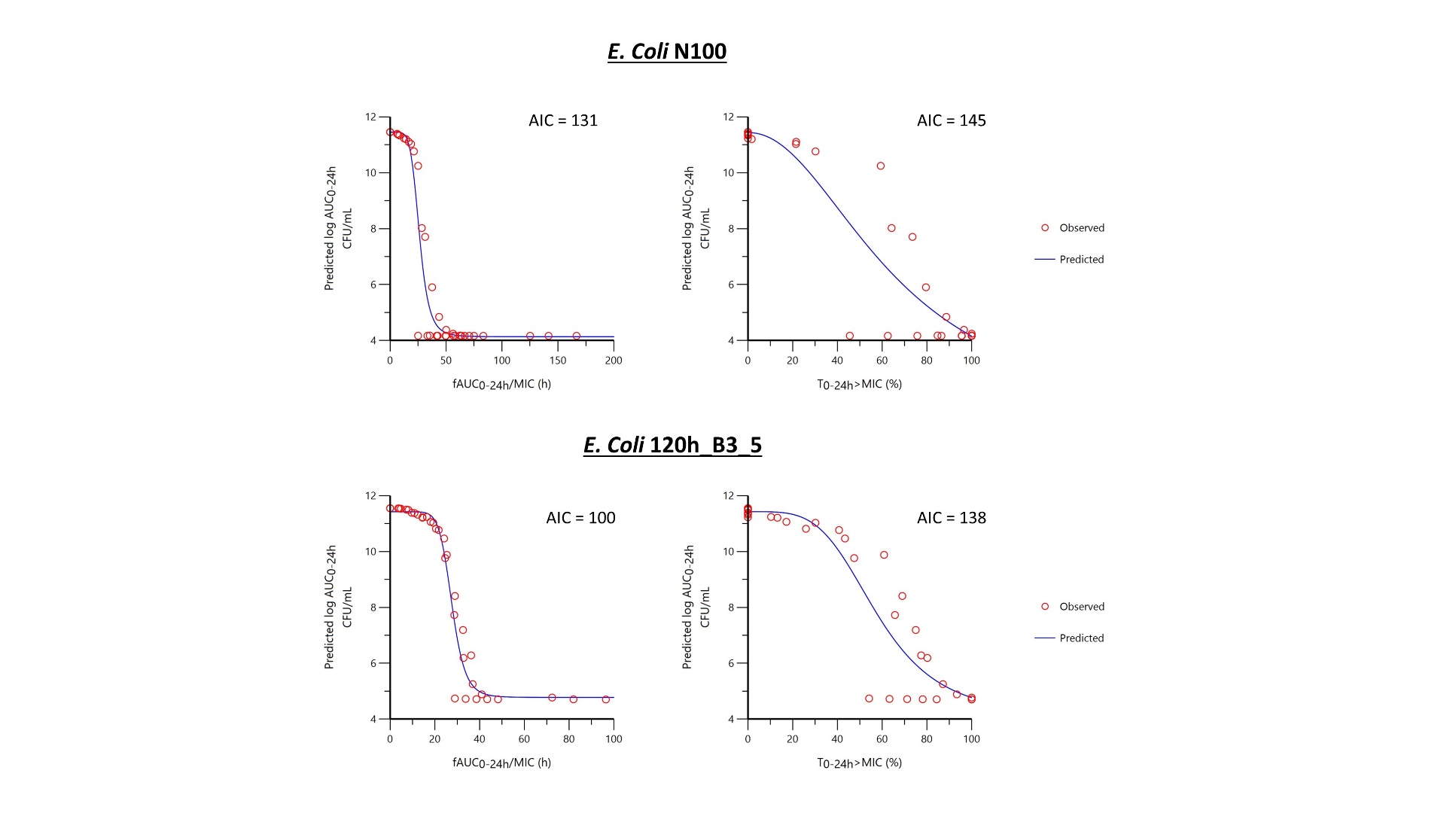


Supplementary Figure 5: Semilogarithmic plot of the prediction of bacteriological effects estimated by the log 10 of the AUC of the bacterial population measured over 24h i.e. AUC_bact(0-24h)_ as a function of the selected PK/PD index, i.e. *f*AUC_PK(0-24h)_/MIC or T>MIC. In the absence of colistin, AUC_bact(0-24h)_ is maximal and equal to 11.4 h and a log10 of AUC_bact(0-24h)_ equal or lower than 4x indicates that colistin was able to eradicate the pathogen with cfu counts that fell below the limit of quantification of the analytic method i.e. 100 cfu/mL. Data were fitted with an I_max­_ sigmoid model with *f*AUC_PK(0-24h)_/MIC or T>MIC as a predictive variable and log­_10_ AUC_bact(0-24h)_ as dependent variable for *mcr*-negative isolate N100 and *mcr*-positive isolate 120h_B3_5 with fraction of free colistin at 0% protein binding. A reduction of 1 log cfu over 24 h corresponds to a log10 AUC_bact(0-24h)_ of 6.15.
